# The Role of the Gut Microbiome, Immunity, and Neuroinflammation in the Pathophysiology of Eating Disorders

**DOI:** 10.3390/nu13020500

**Published:** 2021-02-03

**Authors:** Michael J. Butler, Alexis A. Perrini, Lisa A. Eckel

**Affiliations:** 1 Institute for Behavioral Medicine Research, Ohio State University Wexner Medical Center, Columbus, OH 43210, USA; Michael.Butler@osumc.edu; 2 Department of Psychology and Program in Neuroscience, Florida State University, Tallahassee, FL 32306, USA; perrini@psy.fsu.edu

**Keywords:** anorexia nervosa, bulimia nervosa, binge eating, cytokines, gut dysbiosis

## Abstract

There is a growing recognition that both the gut microbiome and the immune system are involved in a number of psychiatric illnesses, including eating disorders. This should come as no surprise, given the important roles of diet composition, eating patterns, and daily caloric intake in modulating both biological systems. Here, we review the evidence that alterations in the gut microbiome and immune system may serve not only to maintain and exacerbate dysregulated eating behavior, characterized by caloric restriction in anorexia nervosa and binge eating in bulimia nervosa and binge eating disorder, but may also serve as biomarkers of increased risk for developing an eating disorder. We focus on studies examining gut dysbiosis, peripheral inflammation, and neuroinflammation in each of these eating disorders, and explore the available data from preclinical rodent models of anorexia and binge-like eating that may be useful in providing a better understanding of the biological mechanisms underlying eating disorders. Such knowledge is critical to developing novel, highly effective treatments for these often intractable and unremitting eating disorders.

## 1. Introduction

Eating disorders are serious psychiatric conditions driven, in part, by non-homeostatic eating, including chronic underconsumption of calories in anorexia nervosa (AN) and intermittent binge eating in bulimic syndromes including binge eating disorder (BED) and bulimia nervosa (BN). While AN is often defined by chronic caloric restriction, other core features include increased physical activity, an intense fear of weight gain, endocrine alterations, disturbance of body image, and low body weight [1]. This eating disorder disproportionately affects females with a 12-month prevalence of 0.4–1% among adolescents and young adults [1]. In addition to environmental factors, such as the cultural pressure for thinness in women, it is clear that genetic and hormonal factors also play an important role in the development and maintenance of AN [2,3,4]. Consistent with its complex and poorly understood etiology, the course of AN is highly variable, relapse is common, and the mortality rate is the highest among psychiatric illnesses [5,6]. This highlights a pressing need for research designed to better understand the biological mechanisms underlying AN and to identify biological risk factors.

At the other end of the spectrum, bulimic syndromes are characterized by binge eating, which involves the recurrent consumption of objectively large amounts of food in a discrete period of time that is accompanied by a loss of control over eating [7]. Intermittent binge eating is a core feature of multiple bulimic syndromes, including BED, BN, and the binge–purge subtype of AN. As such, a greater understanding of the biological mechanisms underlying binge eating in particular could advance the identification, prevention, and treatment of a range of eating disorders. While bulimic syndromes share the core feature of binge eating, BED is unique in that recurrent episodes of binge eating are not accompanied by purging behavior [7], and bingeing in the absence of purging promotes weight gain resulting in an overweight or obese phenotype in individuals diagnosed with BED. In comparison, binge eating in individuals diagnosed with BN or binge–purge AN are accompanied by compensatory behaviors, including self-induced vomiting, excessive exercise, laxative and diuretic abuse, and prolonged fasting [8]. Collectively, these compensatory behaviors often mitigate the excess calories consumed during a binge and thus serve to constrain weight gain. Of course, these compensatory behaviors can lead to other complications, including sialadenosis, acid-base and electrolyte changes, hypokalemia, and disturbances in upper gastrointestinal function [9,10]. In studying the biological mechanisms underlying binge eating, it is important to recognize that this form of dysregulated eating behavior occurs along a body mass index (BMI) continuum ranging from underweight to obese.

Similar to AN, highly effective treatments for bulimic syndromes, including BN and BED, are lacking [11], and efforts to develop effective treatments are hampered by our limited understanding of the biological mechanisms underlying these eating disorders. The last few years have, however, led to a growing recognition that both the gut microbiome and the immune system are involved in a number of psychiatric illnesses, including eating disorders [11,12]. Moreover, the important roles that diet composition and caloric intake play in terms of regulating microbiome and immune function has spurred further interest in studying these biological systems in the context of dysregulated eating behavior. Here, we review the evidence that alterations in the microbiome and immune function can promote and maintain eating disorders characterized by caloric restriction in AN and intermittent binge eating in bulimic syndromes including BN and BED.

## 2. Eating Disorders and the Gut Microbiome

The gut microbiome has become an emerging focus in a number of health disciplines, including the diagnosis and treatment of psychiatric disorders [13]. This should come as no surprise given that the human body contains at least as many prokaryotic bacterial cells as total eukaryotic cells [14,15]. Moreover, the human microbiome contains 150 times more bacterial genes, derived from thousands of gut microbial species, than what exists in the human genome [14]. The collective genomes of all the microbes lining the full length of the gastrointestinal tract comprise the “gut microbiome”.

A common metric of gut health is the diversity of microbial species that inhabit it. Alpha diversity is represented by the variation of microbes within a sample, and is a good indicator of species richness, which is determined by the number of different species of microbes present in a given fecal/cecal sample [16]. In general, a more diverse population of gut microbes is associated with better health outcomes. Conversely, low alpha diversity, together with an increased Firmicutes: Bacteriodetes ratio, is associated with increased gut permeability or a “leaky gut”, which has been causally linked to gastrointestinal problems, inflammation, and various disease states [13]. This symbiotic relationship between gut microbes and their host affects everything from host metabolism and body weight regulation to endocrine, immune, and nervous system function [13].

Since the ecosystem of gut microbes is constantly interacting with ingested nutrients and the eukaryotic cells that comprise the intestinal tissue and line the gut wall [13], both short- and long-term dietary changes can affect the composition and abundance of gut microbiota [17]. Recognition of the important reciprocal interplay between diet and gut microbes, together with the well-defined role of the microbiome in regulating metabolic function and body weight [17], has spurred recent interest in studying the microbiome in patients with eating disorders.

Several systematic reviews of the obesity and microbiome literature highlight substantial differences in gut microbial composition between lean and obese individuals [17,18]. As mentioned above, diversity of gut microbes is a key indicator of gut health. As such, several studies have reported a reduction in microbial alpha diversity in obese individuals compared to lean individuals. Specifically, a reduction in butyrate-producing bacteria has been associated with obesity in humans [19]. Butyrate is a short-chain fatty acid and a bacterial byproduct that is a consequence of fiber-metabolizing bacteria in the colon [20]. Butyrate and other short chain fatty acids are important anti-inflammatory mediators in both the gut and the brain, and their deficiency is associated with several chronic diseases, including metabolic disorders, obesity, and type 2 diabetes [20].

Studies involving manipulation of the microbiome further highlight its important role in regulating body weight. For example, the use of prebiotics and probiotics can reduce body weight and fat mass in obese individuals [21], and fecal microbiome transplants from healthy donors has been shown to improve glucose metabolism, insulin sensitivity, and liver function in overweight individuals [22,23]. It has also been shown that fecal microbiome transplants from obese individuals promotes weight gain in germ-free, lean rodents despite no change in caloric intake [24,25]. Taken together, these studies provide compelling evidence that the gut microbiome plays a critical role in regulating body weight and thus offers an important therapeutic target for slowing or reversing weight gain in obese individuals.

### 2.1. The Microbiome and Anorexia Nervosa

While much is known about the role of the microbiome in the development and maintenance of obesity, far fewer studies have been conducted in individuals with eating disorders and, to the best of our knowledge, this emerging literature is restricted to AN. In the first comprehensive investigation, Kleiman et al. [16] characterized the fecal microbiome of a small group of AN patients upon admission (*n* = 16) and again at discharge (*n* = 10) from an inpatient treatment facility. At admission, acutely ill AN patients had significantly reduced alpha diversity, relative to healthy controls. Interestingly, alpha diversity was associated with eating disorder psychopathology, with reduced bacterial species associated with greater depression and concern about weight and shape [16]. While the health of the fecal microbiome improved in weight-restored AN patients, alpha diversity continued to be significantly reduced in the AN group at discharge, and significant difference in beta diversity (a measure of how samples of the microbiome vary against each other) were also observed in AN patients between admission and discharge [16]. This is particularly interesting as it suggests that alterations in the gut microbiome may be trait related and thus may play a causal role in AN, rather than simply being a consequence of malnutrition or low body weight.

Most, but not all, studies investigating the fecal microbiome in AN have reported some level of gut dysbiosis, defined by bacterial colonies in the gastrointestinal tract that are disrupted in a way that is detrimental to the host [26,27,28,29,30]. While some studies replicated the first report of decreased alpha diversity in acutely ill AN patients [31,32], others have not [27,28,33]. In light of the strong association between alpha diversity and overall gut health, these equivocal findings highlight the need for additional investigations of alpha diversity in the gut microbiome of individuals diagnosed with, and recovered from, AN. Further research is also needed to assess the specific changes in microbial composition in AN, including changes in beta diversity and microbial metabolites that may perpetuate AN symptoms.

### 2.2. The Microbiome in Animal Models of Anorexia Nervosa

Preclinical rodent models of AN have been valuable in terms of studying the biological mechanisms underlying this eating disorder [34,35,36] and should be particularly useful in elucidating how gut dysbiosis may serve as a biomarker for the development or maintenance of AN. Activity-based anorexia (ABA), one of the more common rodent models of AN, combines food restriction (e.g., limiting food access to 1–2 h per day or providing 40% of baseline food intake) with the opportunity to exercise in a running wheel [37,38]. Under these conditions, rodents eat less than sedentary controls given the same restricted access to food and engage in high levels of running wheel activity, resulting in rapid weight loss. In addition to the caloric restriction and hyperactivity, ABA-exposed rodents develop other symptoms of AN, including low adiposity, hypothermia, and neuroendocrine disruptions [37]. Given the complex social and psychiatric components of AN, it is clear that the ABA paradigm can only model a subset of AN symptoms, but it is widely recognized as the best preclinical model of this complex eating disorder [34,39].

A small but growing number of studies have examined the impact of ABA on the gut microbiome. A proteomic analysis of fecal samples collected from male mice exposed to the ABA paradigm revealed increases in multiple *Clostridium* species belonging to the order Clostridiales, and an increase in phosphoglycerate kinase, a glycolytic ATP-producing enzyme, relative to freely-fed and food restricted/sedentary control mice [40]. Despite these adaptive changes to the negative energy balance seen in ABA mice, in vitro assays revealed no reliable differences in the ATP-producing capacity of bacterial protein from ABA mice, relative to control mice [40]. A gene sequencing and quantitative PCR analysis of cecal samples collected from male mice with ABA revealed increases in the abundance of *Clostridium* and *Lactobacillus* species and a decrease in the abundance of *Burkholderiales* species, relative to freely-fed mice [41]. This group also showed that most of these changes in the gut microbiome could be attributed to food restriction/starvation as opposed to the increased activity levels observed in ABA mice [41]. Gut dysbiosis has also been reported in male rats with ABA, but changes in their fecal microbiome included decreased abundance of Firmicutes, Bacteroidetes, and Lactobacillus, and an increase in *M. smithii*, in comparison to freely-fed rats [42]. These differing profiles of gut microbiota alterations in mice and rats may reflect a species difference and/or methodological differences related to sample collection, duration of food access, and length of the ABA paradigm [41]. In one of the few studies to examine the impact of ABA on the microbiome of female rodents, Trinh et al. [43] reported differences in beta diversity between rats with and without ABA, and an increase in alpha diversity in ABA rats that was negatively correlated with global brain weight. While an increase in alpha diversity typically signals better health, the authors speculated that this increase in alpha diversity in ABA rats may be an adaption to starvation [43], but this requires further testing.

While there is compelling evidence of gut dysbiosis in individuals diagnosed with AN, rodents with ABA, and rodents subjected to chronic food restriction [44], much less is known about the functional consequence or molecular underpinnings of the altered microbiome. In a recent study by Dominique et al. [45], male mice with ABA were reported to have elevated levels of a gut microbial protein that may mimic the effects of alpha-melanocyte stimulating hormone (α-MSH), an anorexigenic neuropeptide that plays a critical role in regulating appetite. Additional studies are needed to assess whether this particular gut microbe, caseinolytic peptidase B, is also elevated in AN and whether it may act to promote or maintain calorie restriction. In another recent study, the fecal microbiome of four individuals with AN and four healthy controls was transferred to germ-free mice, and alterations in weight gain and behavior were measured [46]. Mice receiving microbes from AN patients displayed a reduction in food intake and impaired weight gain relative to mice receiving microbes from healthy controls [46]. The mice receiving fecal microbiome transplants from individuals with AN also displayed increased anxiety- and compulsive-like behavior relative to controls [46]. This suggests that gut dysbiosis is a critical mediator of AN pathology and thus could be targeted directly to improve AN symptoms.

In support of this view, fecal microbiome transplantation has been used in AN patients with promising results. In one case study, an AN patient with significant impairments in the gut barrier function and low alpha diversity showed improvements in both measures following the fecal microbiome transplant from a healthy, first-degree relative [47]. Short chain fatty acids were also increased following fecal microbiome transplant, as was serotonin [47]. In another study, a patient with AN showed significant improvement in weight gain following fecal microbiome transplant from a non-related healthy female donor [48]. This increase in weight gain was driven mostly by a 55% increase in body fat despite a reported stable caloric intake [48]. The ability of fecal microbiome transplants to increase body weight/adiposity without a concomitant increase in food intake has significant treatment implications for those suffering from severe AN, as refeeding is often difficult in these populations. Given that these are case reports and no large-scale, randomized controlled trials haven been used to assess the impact of fecal microbiome transplant in AN, these findings should be interpreted with some caution. Nevertheless, they provide a proof-of-concept that treating gut dysbiosis in AN may be a promising therapeutic approach.

Decreased alpha diversity and other markers of an unhealthy microbiome have been shown to increase gut permeability, which can promote gastrointestinal problems and inflammation [13]. Consistent with these findings, histological analysis of the gut in underweight male mice with ABA revealed increased permeability of the colon, decreased gastric wall thickness, and decreased expression of tight junction proteins [49]. While similar direct measures of gut permeability cannot be conducted in humans, indirect assessments involving non-invasive methodologies are possible but have yielded equivocal findings. In one study, a reduction in gut permeability was reported in AN patients following assessment of mannitol and lactulose absorption from the small intestine [50]. However, a more recent pilot study by Mörkl at al. [32] reported no changes in gut permeability between AN patients and healthy controls following assessment of plasma zonulin, a protein that modulates the permeability of tight junctions between intestinal cells. While neither of these studies provide evidence of increased gut permeability in AN, similar to that reported in the preclinical ABA model [49,51], conclusions are limited by the small number of AN patients examined to date.

In addition to altering colon permeability, the gut microbiome has been shown to impact gut mucosal barrier function [13]. This raises the possibility that, in AN, the integrity of the gut mucosal layer could be compromised by an increase in certain bacteria that preferably digest proteins, including those of the intestinal mucus, leading to increased colonic permeability in AN. In support of this notion, human studies have shown an increase in the number of mucin-degrading bacteria in the gut of individuals diagnosed with AN [16].

A summary of the changes in the gut microbiome in AN and animal models of AN is provided in Table 1.

## 3. Anorexia Nervosa and Peripheral Inflammation

The immune system is comprised of two branches, the innate and the adaptive immune system, which play a coordinated role in detecting and neutralizing invading bloodborne and cellular pathogens [52]. The innate immune system is usually the first to respond to an invading pathogen and rapidly produces a generalized, non-specific inflammatory response mediated by neutrophils and eosinophils, followed by macrophages. The B and T lymphocytes of the adaptive immune system respond more slowly but the response is specific to the pathogen and can be recalled upon re-exposure to the pathogen [52].

As reviewed above, alterations in the gut microbiome in AN may increase gut permeability via decreased intestinal mucins, thinning of the intestinal walls, and altered tight junctions, all of which can facilitate the diffusion of bacterial byproducts and, often times, whole bacteria across the intestinal wall barrier and into the bloodstream [13]. This rise in bacteria and/or bacterial byproducts could activate immune cells and trigger peripheral inflammation. This type of immune response elicited by gut dysbiosis is likely initiated via activation of toll-like receptors (TLRs) [53]. TLRs are cell surface receptors that can directly bind bacterial pathogens and byproducts and are expressed on the membranes of multiple immune cell subtypes, including macrophages, neutrophils, and dendritic cells of the innate immune system. The binding of TLRs, such as TLR4, by bacteria and their metabolites activates the innate immune system, triggering canonical inflammatory signaling pathways and transcription factors, such as nuclear factor kappa B (NF-kB), that increase the transcription of proinflammatory cytokines.

Initial studies involving clinical populations of individuals diagnosed with AN provide evidence of peripheral immune system impairment. For example, a small study involving 10 AN patients reported significantly decreased neutrophil chemotaxis in comparison to a larger group of healthy controls [54]. Another study showed a reduction in the ability of granulocytes, isolated from a small AN sample, to kill two different bacterial species [55]. This same study also reported evidence of decreased neutrophil phagocytosis, which is consistent with decreased chemotaxis [55]. In addition to these early studies, two recent meta-analyses of cross-sectional and longitudinal clinical studies support the notion that AN is associated with chronic, low-grade inflammation. This inflammatory phenotype is most consistently characterized by increased serum/plasma levels of proinflammatory cytokines such as interleukin 1 beta (IL-1β), interleukin 6 (IL-6), and tumor necrosis factor alpha (TNF-α) [56,57]. For example, peripheral blood mononuclear cells (PBMCs), isolated from patients with AN, were shown to have increased spontaneous production of TNF-α in comparison to similarly isolated PBMCs from healthy controls [58]. This increased production of TNF-α was normalized upon refeeding [58]. This finding was replicated in another study showing an increase in spontaneous proinflammatory cytokine release from PBMCs isolated from AN patients that normalized upon weight gain [59]. The refeeding reversal of immune activity reported in these studies suggests that nutritional/starvation status plays a direct role in the peripheral immune response in AN.

Circulating levels of proinflammatory cytokines have also been studied in AN patients. An exploratory cross-sectional study that examined serum concentrations of multiple markers of inflammation in acutely ill AN patients revealed an increase in IL-6, IL-15, and vascular cell adhesion molecule (VCAM)-1, relative to healthy controls [60]. More recently, a longitudinal study reported an increase in plasma IL-6 in acutely ill AN patients that normalized, along with AN symptoms, after 12 weeks of treatment [61]. Taken together, these findings suggest that elevated proinflammatory cytokines, and IL-6 in particular, may serve as state markers for AN. Moreover, a recent meta-analysis revealed that selective inhibition of the IL-6 signaling pathway is associated with an increase in weight gain [62]. This suggests that the IL-6 signaling pathway is involved in the regulation of body weight and could thus be targeted by available pharmacological IL-6 inhibitors to facilitate weight gain in AN patients.

Adaptive immunity also appears to be impaired in AN. T cell subtypes are dysregulated in AN patients when compared to healthy controls and to individuals with primary malnutrition [63,64,65,66]. The CD4/CD8 T cell ratio is increased in AN patients and this is likely due to a greater reduction in CD8 counts compared to CD4 counts. Multiple studies have suggested this reduced CD8 cell count is driven by a reduction in memory CD8 cells as opposed to naïve CD8 cells [65,66]. Indeed, these studies suggest that with the significant decrease in body weight, lymphocyte production, specifically T helper cells (CD4 cells), is favored over other lymphocytes in an effort to preserve the efficacy of the adaptive immune system [52].

Fewer studies have looked at B cell function in AN patients and the available data are equivocal. One study found normal serum levels of immunoglobin A (IgA), IgM, and IgG antibodies (produced by B cells) in 16 AN patients [52], while a smaller study involving only five AN patients found lower levels of IgM and IgG antibodies in AN patients compared to healthy controls [67]. Interestingly, detectable differences in B cell function may only be present in the most severe cases of AN (BMI less than 17.5) as IgG was reduced in these patients compared to healthy controls [59]. Moreover, in a larger study of AN patients (*n* = 46), B cell count was negatively correlated with BMI, and T and B cell interactions were also dysregulated [65]. Overall, while large-scale studies in this area are lacking, it is clear that AN is associated with multiple abnormalities of the immune system in addition to increased systemic inflammation and inflammation of peripheral tissues.

Consistent with impairments in innate and adaptive immunity in AN patients, there is some evidence that these changes in immune system function may exacerbate the complications of infectious disease although, aside from a few studies and case reports, data in this area are lacking. In one study, an attenuated fever response and delayed immune reaction was associated with increased complications from bacterial infections in AN patients compared to healthy controls [68]. Conversely, an examination of case studies revealed that AN patients report fewer symptomatic viral infections than controls, and the viral infection risk appears to increase as AN patients gain weight during recovery [69]. It is unclear what immunological mechanisms may protect AN patients from viral infection. One possibility is that AN patients may present with fewer viral symptoms but actually have higher viral titers than healthy controls, as is the case with multiple sclerosis, but studies testing this hypothesis in AN are lacking [69].

Data from rodent models of AN, specifically the ABA model described above, may shed some light on the underlying mechanisms of impaired peripheral immune function in AN. Increased levels of proinflammatory cytokines have been observed in ABA. For example, ABA mice displayed early onset intestinal inflammation marked by increased levels of TLR4 mRNA in the colon and an increase in the cell surface expression of TLR4 on colonic macrophages and epithelial cells [70]. ABA was also associated with elevated levels of mucosal proinflammatory cytokine production, specifically TNF-α and IL-6 [70]. It is important to note that these same inflammatory changes were observed in a limited food access control group as well, suggesting chronic malnutrition/starvation alone is sufficient to alter immune function in the mouse gut. In the same study, TLR4 knockout mice had higher rates of mortality due to ABA than wildtype controls, suggesting that while TLR4 may be integral to the proinflammatory response observed in ABA, it also appears to be playing a protective role. However, the underlying mechanisms of TLR4′s protective effects in the context of ABA are unknown and require further investigation [70].

## 4. Anorexia Nervosa and Neuroinflammation

It is now widely accepted that peripheral inflammatory events can directly cause inflammation in the central nervous system. Circulating proinflammatory cytokines have been shown to cross the blood brain barrier and stimulate the release of proinflammatory cytokines from microglia, which express TLRs and serve as the resident immune cells of the brain [71]. Microglia are myeloid cells derived from erythromyeloid progenitors in the yolk sac and enter the nervous tissue early during embryonic development [72]. Central changes in immune signaling can have significant impacts on a variety of brain functions, including feeding behavior and mood regulation [71,73], both of which are dysregulated in AN. While the role of neuroinflammation in AN patients is unknown, evidence from a number of preclinical rodent models suggest that central inflammation, and specifically microglia, play an important role in the pathophysiology of AN.

Brain concentrations of proinflammatory eicosanoids derived from the omega-6 polyunsaturated fatty acid (PUFA) arachidonic acid were increased in rats at the induction of, and recovery from, the ABA phenotype, relative to exercise and food-restricted controls [74]. Eicosanoids are bioactive metabolites of PUFAs that serve a variety of physiological and pathological functions in the nervous system, including regulating immune function [75]. While inflammation (i.e., cytokine expression) was not directly assessed in this study, the authors did show significant increases in proinflammatory arachidonic acid metabolites including 5(S)-hydroxyeicosatetraenoic acid (5(S)-HETE), 8(S)-HETE, 12(S)-HETE, 15(S)-HETE, and several prostaglandins in a brain-region specific manner [74]. These metabolites serve as natural ligands to receptors expressed on the surface of microglia and can directly modulate microglial activity [75]. It is likely that this ABA-induced alteration of PUFA metabolism does, indeed, alter microglial activity and increase neuroinflammation, but this hypothesis remains to be tested in future studies. Furthermore, it would be interesting to investigate whether or not ABA also alters omega–3 PUFA metabolism, which competes for the same metabolic enzymes as arachidonic acid but produces anti-inflammatory eicosanoids [75].

Microglia have been directly implicated in other rodent models of AN. In a dehydration-induced anorexia (DIA) model, which recapitulates weight loss and food avoidance despite its availability, microglial density and morphological indicators of activation were increased in the prefrontal cortex, relative to controls [76]. This cellular response was accompanied by an increase in proinflammatory cytokines such as IL-1β, IL-6, and TNF-α, confirming a proinflammatory environment in the prefrontal cortex in DIA animals [76]. Interestingly, in this same study, the authors included a forced food restriction control group without the dehydration component, and saw similar results in regards to microglial density, morphology, and proinflammatory markers [76]. These data suggest that severe caloric restriction and nutrient deprivation alone can lead to neuroimmune alterations in the prefrontal cortex. In another study conducted by this same group, DIA led to similar results in the hippocampus as it did in the prefrontal cortex, suggesting that multiple brain regions are affected. Given the profound eating pathology in AN, it will be important to examine microglial expression in additional brain areas that process feeding and reward in this and other animal models of AN. While the role of microglia and other proinflammatory factors are unknown in AN patients, rodent models of AN have shown that neuroinflammation can lead to neuronal loss [76]. Importantly, a decrease in hippocampal volume and structural alterations in the prefrontal cortex have also been reported in patients diagnosed with AN [77,78,79,80,81]. These rodent models suggest that this decrease in hippocampal volume in AN may be due to sustained neuroinflammation.

Microglia and neuroinflammation have also been implicated in the *anx/anx* mouse model of AN. This model arose over 40 years ago from a spontaneous mutation, though the exact mapping of the mutation is still unknown [82]. These *anx/anx* mice display the core features of AN-starvation and emaciation in the presence of food accompanied by premature death. They appear normal at birth and are indistinguishable from their wild-type littermates but, despite full access to their mother’s milk, eat significantly less than wild-type mice and die at 3–5 weeks of age [82]. The brains of these *anx/anx* mice show significant alterations in feeding-related neuropeptides in the arcuate nucleus of the hypothalamus (ARC), including agouti gene-related protein (AgRP), neuropeptide Y (NPY), and proopiomelanocortin (POMC) [82,83]. These alterations are accompanied by increased microglial density and activation in the hypothalamus, and neurodegeneration of hypothalamic neurons that control appetite [84]. While hypothalamic microglia do mediate the neuronal response to satiety signals [73], it is unclear if they directly mediate the alterations of ARC neuropeptides in *anx/anx* mice. Given that there is limited research investigating microglial function in AN rodent models, such as ABA, future studies should investigate the causal relationship between microglia and neuronal function in the hypothalamus. Hypothalamic function also appears to be altered in AN patients, as several recent studies have shown individuals with AN have hypothalamic structural alterations [81], decreased hypothalamic connectivity to the striatum [85], and impaired hypothalamic glucose reactivity compared to healthy-weight controls [78].

Taken together, the rodent literature indicates significant neuroinflammation across multiple brain regions that control food intake and regulate bodyweight and mood, all of which are disrupted in AN. However, with limited availability of postmortem brains from AN patients it makes it difficult to illuminate the role of microglia and neuroinflammation in the pathology of AN. Given what we know from multiple animal models, coupled with our current understanding of nervous and immune system interactions, it is likely that neuroinflammation is playing a role in AN pathology. Future studies could focus on utilizing imaging methods of gliosis [86] or positron emission tomography (PET) imaging of microglia-specific markers [87] in AN clinical populations to better understand the role of neuroinflammation in the development and maintenance of this eating disorder.

A comprehensive summary of the changes in peripheral and central immune function in AN and animal models of AN is provided in Table 2.

## 5. Bulimic Syndromes and Inflammation

A shared feature of bulimic syndromes is binge eating, a form of dysregulated eating behavior involving intermittent overconsumption of palatable foods that are high in saturated fats and refined sugar [88]. It is well-documented that chronic consumption of a high fat diet is associated with peripheral and central inflammation, both of which contribute to weight gain and obesity-related comorbidities including metabolic syndrome, insulin resistance, and type 2 diabetes. While there is a robust literature examining immune function in individuals diagnosed with AN, much less is known about the immune system’s response to intermittent overconsumption of calories in individuals diagnosed with bulimic syndromes, including BN and BED, or whether dysregulated immune function may serve as a risk factor for either of these eating disorders.

Proinflammatory cytokines, such as IL-1β, IL-6, and TNF-α, can act directly on the neuronal circuits that control food intake in the hypothalamus [89]. In comparison to the AN literature, far fewer studies have examined whether bulimic syndromes are associated with elevated levels of proinflammatory cytokines. The first exploratory study to examine plasma cytokines in a small sample of 20 BN patients reported elevated levels of TNF-α in comparison to healthy controls [90]. Similar results were reported in another study in which plasma levels of IL-1β and thiobarbituric acid reactive substances (TBARS; a marker of systemic oxidative stress) were found to be elevated in a mixed sample of eating disorder patients (60% were diagnosed with BN or BED), relative to healthy controls [91].

In contrast to these studies, a recent meta-analysis, which excluded the aforementioned studies due to small sample size and the heterogeneous eating disorder group, examined the findings of four studies that measured circulating proinflammatory cytokine concentrations in BN patients. Their analysis revealed no difference in IL-6 or TNF-α in individuals with BN compared to healthy controls [56]. The same meta-analysis, which searched for studies dating back to the early 1980s, failed to identify any studies examining proinflammatory cytokine concentrations in individuals diagnosed with BED. These findings should be interpreted with some caution, however, as most of the studies in the meta-analysis failed to account for confounding factors that could affect cytokine production, comorbid diagnoses related to physical and psychiatric health, differences in treatment regimens, and differences in the duration or severity of BN illness [56]. Since the publication of this meta-analysis, a recent study reported increased serum levels of IL-1β, IL-6, and TNF-α in a large group of BN patients (*n* = 76), compared to healthy controls [92]. It should be noted, however, that all of the BN participants in this study were obese (average BMI >40) and undergoing evaluation for bariatric surgery. As such, the increase in proinflammatory cytokines may be entirely related to the excess adiposity in this sample of BN patients.

In addition to assessing the concentration of proinflammatory cytokines circulating in the blood, in vitro assays can be used to assess the production and release of proinflammatory cytokines in PBMCs. To the best of our knowledge, only a single study has examined cytokine production in a bulimic syndrome and they reported no difference in either unstimulated or stimulated production of IFN-gamma, IL-1β, IL-6, or TNF-α from PBMCs in individuals diagnosed with BN, in comparison to healthy controls [93].

While the available (but scant) literature suggests proinflammatory cytokines do not appear to be systematically altered in BN in a way that is observed in AN, it has been shown that plasma concentration of IL-10, an anti-inflammatory cytokine [94], is significantly lower in obese individuals diagnosed with BED, in comparison to healthy controls [95]. In this same study, it was reported that plasma IL-10 concentration was also decreased in obese individuals without BED or any other eating pathology [95], so this alteration in circulating levels of IL-10 may be driven solely by the elevated adiposity in obese individuals with and without a diagnosis of BED. To better understand the functional significance of this finding, it will be important for future studies involving BED patients to examine circulating levels of proinflammatory cytokines in general, and TNF-α in particular, as IL-10 has been shown to regulate expression of the TNF-α converting enzyme [96]. Since IL-10 is involved in regulating both the innate and adaptive immune response [94,97], additional studies are needed to further clarify the role of IL-10 and other anti-inflammatory cytokines in bulimic syndromes.

Beyond measuring circulating levels of proinflammatory or anti-inflammatory cytokines, other biomarkers of inflammation and associated compromised metabolic health have been examined in the context of bulimic syndromes. For example, one study found that loss of control eating in adolescents was associated with increased concentrations of high-sensitive C-reactive protein (hs-CRP), relative to healthy controls [98]. This finding was replicated and extended by another group, which found that obese individuals with BED had a poorer inflammatory profile, as measured by elevated levels of hs-CRP and white blood cells, in comparison to obese individuals without a diagnosis of BED [99]. Another study reported lower levels of adiponectin, an adipokine that promotes metabolic health, in BED-obese individuals in comparison to obese individuals without BED [100]. The findings of the latter two studies are particularly interesting as they suggest a specific link between inflammation and intermittent binge eating that is not simply the result of being overweight and thus may be driven by dysregulated eating behavior. While the mechanism underlying such a relationship is unclear, it has been shown that intermittent binge eating and the rapid rate of eating in BED has greater adverse metabolic consequences than the chronic overeating of smaller but more frequent meals in non-BED obese individuals, and this may serve to compromise immune function [101,102,103].

Interestingly, a primary deficit in T-cell levels has also been reported in BN patients. In one study involving patients with AN, BN, and healthy controls, the CD4/CD8 T-cell ratio was found to be significantly lower in the BN group, relative to the AN group, with both eating disorder groups having lower CD4/CD8 ratios than healthy controls [104]. Since the CD4/CD8 T-cell ratio is a marker of immune system function, with lower ratios associated with greater risk of immunodeficiency and autoimmune disease, these findings suggest greater inflammation in the BN group, relative to the AN group. However, both eating disorder groups displayed similar results on a cutaneous hypersensitivity assay suggesting that their cell-mediated immune response was similarly compromised [104]. In a follow-up study of BN patients with normal BMI and no signs of malnutrition, a decrease in multiple T-cell types (CD2, CD3, CD4, CD8, and CD57) was found in the BN group, relative to healthy controls [105]. Consistent with their earlier study [104], these findings confirm depleted immunocompetence in the BN group.

A small but growing literature has begun to document an association between inflammatory-based disease and subsequent risk for developing a bulimic syndrome. Most notably, a large, nation-wide study of children and adolescents born in Denmark over a period of 17 years reported that childhood diagnosis of an autoimmune or autoinflammatory disease increased the risk of developing AN by 36% and BN by 73% within this cohort [106]. They also reported that familial autoimmune disease predicts eating disorder risk in offspring [106]. Importantly, this study replicates and extends an earlier report by the same group linking inflammatory disease and eating disorder risk in a Finnish population [107]. Additional support for the notion that inflammation may play a causal role in the development of bulimic syndromes comes from the Avon Longitudinal Study of Parents and Children (ALSPAC), which followed 1502 children from birth to 18 years of age and used medical records to identify factors that predicted the onset of eating disorders. Compared to healthy children, girls and boys who later developed BN or BED had significantly greater body mass index (BMI) percentiles by the age of 6 years, and increased adiposity and a poorer inflammatory profile nearly 8 years before eating disorder diagnosis [108].

While multiple studies have reported that autoimmune and inflammatory diseases are associated with increased risk for developing BN and BED, the mechanism underlying this relationship is unknown. Since proinflammatory cytokines produced by autoimmune diseases can suppress appetite and promote weight loss [109], it is possible that the resulting energy deficit may serve as a trigger for dysregulated eating behavior in general and binge eating in particular. Another possible mechanism arises for human imaging studies, which have shown that disease-related neuroinflammation may alter activity in reward and feeding pathways [80,110], and this could serve to mediate the association between inflammatory disease and eating disorder risk. Additional studies are needed to better understand how immune system disturbance may promote eating disorders in general, and bulimic syndromes in particular. Such information is a crucial first step toward identifying novel biological targets for treating these conditions. In this regard, rodent-based models of binge eating may be particularly valuable as they permit prospective investigations of individual differences in immune function that may alter vulnerability to binge eat, and the ability to directly manipulate and target the immune system in treatment-focused studies.

### What Can Animal Studies Tell Us?

While several well-validated preclinical animal models of binge eating exist, few have been used to examine the role of the immune system in the development or maintenance of binge eating in bulimic syndromes. To the best of our knowledge, there is only one study that used a frustration stress paradigm [111,112] to promote binge-like eating in female rats. In this paradigm, binge eating of a familiar, palatable food is evoked in rats by exposure to three, 8-day cycles of food restriction (food is restricted to 66% of baseline levels on days 1–4 followed by ad libitum feeding on days 5–8) during which they are given access to the palatable food for 2 h/day on days 5–6 and 13–14 of the first two cycles. During the third cycle, the palatable food is presented only on day 24 under the same conditions as in previous cycles (control condition) or after a 15 min “frustration stressor” in which rats can see and smell the palatable food but are prevented from eating it (binge condition). On this final test day, rats subjected to the frustration stressor engage in binge-like eating of the palatable food, relative to control rats [112]. Using this paradigm, Alboni et al. [113] examined changes in gene transcription of inflammatory markers in the hypothalamus of female rats following binge-like or normal consumption of palatable food. While multiple markers were examined, only three were differentially expressed, with lower mRNA levels of IL-18 and its receptor (IL-18R) and higher levels of inducible nitric oxide synthase (iNOS) mRNA detected in the hypothalamus of binge-eating versus control rats [113]. It is possible that this decreased transcription of IL-18/IL-18R may have played a permissive role in the binge-like eating observed in this study, as a lack of IL-18 signaling promotes hyperphagia, weight gain, and related comorbidities in rodents [114,115]. The increased mRNA expression of iNOS in the hypothalamus of binge-eating rats is also of interest, as iNOS production and release is stimulated by proinflammatory cytokines [116] and thus provides some indirect evidence that binge-like eating in rats is associated with hypothalamic inflammation. Additionally nitric oxide (NO) has been shown to regulate the expression of neuropeptides that control food intake [117] and clinical studies have shown that NO production is increased in individuals with BN [118]. While these preliminary findings are interesting, it will be important to determine whether similar changes are observed in other animal models of binge-like eating in which animals engage in repeated episodes of binge-like eating. It will also be important to look at more direct measures of neuroinflammation including changes in the number and activation state of microglia in multiple brain areas.

A comprehensive summary of the peripheral and central immune changes in BN, BED, and animal models of binge-like eating is provided in Table 3.

## 6. Conclusions

While it is clear that interactions between the gut microbiome, immune system, and nervous system are involved in the pathophysiology of eating disorders, researchers are only beginning to scratch the surface to uncover the intricate mechanisms through which these systems are intertwined. Our summary of the alterations in proinflammatory cytokines, and other markers of peripheral and central inflammation, strongly suggests that the immune system is dysregulated in both anorexic and bulimic syndromes. While AN appears to be associated with an increase in proinflammatory cytokines, immune dysfunction in bulimic syndromes such as BN and BED appear to involve decreased activity of anti-inflammatory cytokines. Additional evidence from longitudinal clinical studies highlights an interesting association between autoimmune and inflammatory diseases and the risk of developing bulimic syndromes. In many studies, the alterations in immune function do not appear to be simply related to malnutrition in AN and BN or excess adiposity in BED. Although the pathogenesis of the alterations in immune function and responsivity in AN, BN, and BED remain unclear, the use of preclinical animal models, like those described here, offer a valuable tool for dissecting the underlying mechanisms. While these kinds of studies are just starting to emerge, with investigations of the microbiome severely lagging in animal models of binge-like eating, they should prove particularly useful in identifying possible biomarkers for early detection and treatment interventions.

While a pattern of dysbiosis and altered inflammatory profile in eating disorders is emerging, concerns regarding publication bias, together with some reports of inconsistent or contradictory findings that may stem from small sample sizes, warrants some caution when drawing conclusions. Within the clinical literature, some of the variable findings may be accounted for by methodological differences in assessing the microbiome and immune function, a failure to consider confounding factors in the measurement of cytokines and the bacterial composition of the gut, and, many times, small or heterogeneous clinical samples. A primary consideration of the preclinical animal-based studies reviewed here is that most of this research has been conducted in male rodents. This is both problematic, as ovarian hormones can have direct effects on the gut microbiome and the immune system, and troubling, as women are disproportionately affected by eating disorders.

Future clinical studies should continue to explore the changes in the gut microbiome and peripheral immunity in eating disorders as these data are lacking, especially in BN and BED. It will also be critical to make use of available human brain imaging technologies, such as PET imaging, to investigate the role of neuroinflammation in eating disorders. Future clinical research would also benefit from a greater focus on longitudinal designs, including acutely ill and recovered individuals, to determine whether alterations in the microbiome or immune system are state or trait markers of eating disorders. Since preclinical rodent models are able to utilize more invasive techniques to quantify gut, immune, and neural changes in response to dysregulated eating, these studies should focus on mechanisms of gut and immune interactions with the brain, with a particular focus on microglial function. Understanding these cellular and molecular mechanisms will be imperative if we are to improve treatment outcomes in patients diagnosed with eating disorders.

## Figures and Tables

**Table 1:** Examination of the gut microbiome in anorexia nervosa (AN) and animal models of AN.

References	Sample	Microbiome Changes
Kleiman et al., 2015 [16]	AN	Decreased alpha diversity at admission and discharge from treatment facility, significant difference in beta diversity.
Mack et al., 2016 [27]	AN	Increased *Clostridium* species, decreased *Roseburia* species, no change in alpha diversity.
Borgo et al., 2017 [28]	AN	Increased *Enterobacteriaceae* and *M. smithii* species, decreased *Roseburia*, *Clostridium*, and *Ruminococcus* species, no change in alpha diversity.
Mörkl et al., 2017 [31]	AN	Decreased alpha diversity.
Mörkl et al., 2019 [32]	AN	Decreased alpha diversity, no change in gut permeability (no change in plasma zonulin).
Schulz et al., 2020 [33]	AN	Increased alpha diversity.
Breton et al., 2019 [40]	Animal model of AN	Increased *Clostridium* species.
Breton et al., 2021 [41]	Animal model of AN	Increased *Clostridium* and *Lactobacillus* species, decreased *Burkholderiales* species.
Queipo-Ortuño et al., 2013 [42]	Animal model of AN	Decreased *Firmicutes*, *Bacteroidetes*, and *Lactobacillus species*, increased *M. smithii* species.
Trinh et al., 2021 [43]	Animal model of AN	Increased alpha diversity, differences in beta diversity.
Dominique et al., 2019 [45]	Animal model of AN	Increased levels of gut microbial protein (caseinolytic peptidase B).
Prochazkova et al., 2019 [47]	Healthy FMT in AN patient (case study)	Improved gut barrier function and alpha diversity, increased SCFAs and serotonin.
De Clercq et al., 2019 [48]	Healthy FMT in AN patient (case study)	Increased adiposity with no change in caloric intake.
Jésus et al., 2014 [49]	Animal model of AN	Increased colon permeability, decreased gastric wall thickness, decreased expression of tight junction proteins.
Monteleone et al., 2004 [50]	AN	Decreased gut permeability (decreased absorption of mannitol and lactulose).

**Table 2:** Summary of peripheral and central immune changes in anorexia nervosa (AN) and animal models of AN.

References	Sample	Immune Changes
Palmblad et al., 2009 [54]	AN	Decreased neutrophil chemotaxis.
Gotch et al., 1975 [55]	AN	Reduction in granulocyte ability to kill bacteria; decreased neutrophil phagocytosis.
Dalton et al., 2018 [56]; Solmi et al., 2015 [57]	AN	Increased proinflammatory cytokines (IL-6, IL-1β, and TNF-α).
Vaisman et al., 1991 [58]; Allende et al., 1998 [59]	AN	Increased proinflammatory cytokine (TNF-α).
Dalton et al., 2018 [60]	AN	Increased proinflammatory cytokines (IL-6, IL-15) and VCAM-1.
Dalton et al., 2020 [61]	AN	Increased plasma proinflammatory cytokine (IL-6).
Fink et al., 1996 [63]; Mustafa et al., 1997 [64]; Elegido et al., 2017 [65]; Nagata et al., 1999 [66]	AN	Dysregulated T cell subtypes compared to healthy controls and individuals with primary malnutrition.
Wyatt et al., 1982 [67]	AN	Decreased levels of IgM and IgG antibodies.
Belmonte et al., 2016 [70]	Animal model of AN	Increased TLR4 mRNA in colon; elevated mucosal IL-6 and TNF-α production.
Collu et el., 2020 [74]	Animal model of AN	Increased proinflammatory eicosanoids.
Reyes-Ortega et al., 2020 [76]; Nilsson et al., 2008 [83]; Nilsson et al., 2011 [84]	Animal model of AN	Increased neuroinflammation and hypothalamic neurodegeneration.

**Table 3:** Summary of the immune changes in bulimia nervosa (BN), binge eating disorder (BED), and animal models of binge-like eating.

References	Sample	Immune Changes
Nakai et al., 2000 [90]	BN	Increased proinflammatory cytokine (TNF-α).
MacDowell et al., 2013 [91]	Mixed sample of eating disorder patients.	Increased proinflammatory cytokine (IL-1β) and TBARS.
Tabasi et al., 2020 [92]	BN	Increased proinflammatory cytokines (IL-1β, IL-6, and TNF-α).
Caroleo et al., 2019 [95]	BED	Decreased anti-inflammatory cytokine (IL-10).
Succurro et al., 2015 [99]	BED	Increased hs-CRP and white blood cells.
Brandão et al., 2010 [100]	BED	Decreased adiponectin.
Marcos et al., 1993 [104]	AN, BN	Decreased CD4/CD8 T-cell ratio, compared to healthy controls.
Marcos et al., 1997 [105]	BN	Decreased T-cell types (CD2, CD3, CD4, CD8, CD57).
Alboni et al., 2017 [113]	Animal model of binge-like eating.	Decreased IL-18 and IL-18R mRNA, and increased iNOS mRNA.
Vannacci et al., 2006 [118]	BN	Increased nitric oxide production.

## Data Availability

Not applicable.

## Abbreviations

FMT	fecal microbiome transplant
SCFAs	short chain fatty acids
IL-6	interleukin 6
IL-1b	interleukin 1beta
TNF-a	tumor necrosis factor alpha
IL-15	interleukin 15
VCAM-1	vascular cell adhesion molecule
IgM	immunoglobulin M
IgG	immunoglobulin G
TLR-4	toll-like receptor 4
TBARS	thiobarbituric acid reactive substances
IL-10	interleukin 10
hs-CRP	high-sensitive C-reactive protein
IL-18	interleukin 18
IL-18R	interleukin 18 receptor
iNOS	inducible nitric oxide synthase

## References

[1] American Psychological Association (2013). Diagnostic and Statistical Manual of Mental Disorders.

[2] Bulik C., Yilmaz Z., Hardaway A. (2015). Genetics and epigenetics of eating disorders. Adv. Genom. Genet..

[3] Hübel C., Gaspar H.A., Coleman J.R.I., Hanscombe K.B., Purves K., Prokopenko I., Graff M., Ngwa J.S., Workalemahu T., O’Reilly P.F. (2019). Genetic correlations of psychiatric traits with body composition and glycemic traits are sex- and age-dependent. Nat. Commun..

[4] Mikhail M.E., Culbert K.M., Sisk C.L., Klump K.L. (2019). Gonadal hormone contributions to individual differences in eating disorder risk. Curr. Opin. Psychiatry.

[5] Hoang U., Goldacre M., James A. (2014). Mortality following hospital discharge with a diagnosis of eating disorder: National record linkage study, England, 2001–2009. Int. J. Eat. Disord..

[6] Fichter M.M., Quadflieg N. (2016). Mortality in eating disorders—Results of a large prospective clinical longitudinal study. Int. J. Eat. Disord..

[7] Mathes W.F., Brownley K.A., Mo X., Bulik C.M. (2009). The biology of binge eating. Appetite.

[8] De Zwaan M. (2001). Binge eating disorder and obesity. Int. J. Obes..

[9] Hetterich L., Mack I., Giel K.E., Zipfel S., Stengel A. (2019). An update on gastrointestinal disturbances in eating disorders. Mol. Cell. Endocrinol..

[10] Westmoreland P., Krantz M.J., Mehler P.S. (2016). Medical complications of anorexia nervosa and bulimia. Am. J. Med..

[11] Rantala M.J., Luoto S., Krama T., Krams I. (2019). Eating Disorders: An evolutionary psychoneuroimmunological approach. Front. Psychol..

[12] Mason B.L. (2017). Feeding systems and the gut microbiome: Gut-brain interactions with relevance to psychiatric Cocditions. Psychosomatics.

[13] Kelly J.R., Kennedy P.J., Cryan J.F., Dinan T.G., Clarke G., Hyland N.P. (2015). Breaking down the barriers: The gut microbiome, intestinal permeability and stress-related psychiatric disorders. Front. Cell. Neurosci..

[14] Seitz J., Trinh S., Herpertz-Dahlmann B. (2019). The microbiome and eating disorders. Psychiatr. Clin. N. Am..

[15] Tremlett H., Bauer K.C., Appel-Cresswell S., Finlay B.B., Waubant E. (2017). The gut microbiome in human neurological disease: A review. Ann. Neurol..

[16] Kleiman S.C., Watson H.J., Bulik-Sullivan E.C., Huh E.Y., Tarantino L.M., Bulik C.M., Carroll I.M. (2015). The intestinal microbiota in acute anorexia nervosa and during renourishment. Psychosom. Med..

[17] Bouter K.E., van Raalte D.H., Groen A.K., Nieuwdorp M. (2017). Role of the gut microbiome in the pathogenesis of obesity and obesity-related metabolic dysfunction. Gastroenterology.

[18] Castaner O., Goday A., Park Y.M., Lee S.H., Magkos F., Shiow S.A.T.E., Schröder H. (2018). The gut microbiome profile in obesity: A systematic review. Int. J. Endocrinol..

[19] Qin J., Li Y., Cai Z., Li S., Zhu J., Zhang F., Liang S., Zhang W., Guan Y., Shen D. (2012). A metagenome-wide association study of gut microbiota in type 2 diabetes. Nature.

[20] Koh A., De Vadder F., Kovatcheva-Datchary P., Bäckhed F. (2016). From dietary fiber to host physiology: Short-chain fatty acids as key bacterial metabolites. Cell.

[21] John G., Wang L., Nanavati J., Twose C., Singh R., Mullin G. (2018). Dietary alteration of the gut microbiome and its impact on weight and fat mass: A systematic review and meta-analysis. Genes.

[22] Kootte R.S., Levin E., Salojärvi J., Smits L.P., Hartstra A.V., Udayappan S.D., Hermes G., Bouter K.E., Koopen A.M., Holst J.J. (2017). Improvement of insulin sensitivity after lean donor feces in metabolic syndrome is driven by baseline intestinal microbiota composition. Cell Metab..

[23] Witjes J.J., Smits L.P., Pekmez C.T., Prodan A., Meijnikman A.S., Troelstra M.A., Bouter K.E.C., Herrema H., Levin E., Holleboom A.G. (2020). Donor fecal microbiota transplantation alters gut microbiota and metabolites in obese individuals with steatohepatitis. Hepatol. Commun..

[24] Ridaura V.K., Faith J.J., Rey F.E., Cheng J., Duncan A.E., Kau A.L., Griffin N.W., Lombard V., Henrissat B., Bain J.R. (2013). Gut microbiota from twins discordant for obesity modulate metabolism in mice. Science.

[25] Turnbaugh P.J., Ley R.E., Mahowald M.A., Magrini V., Mardis E.R., Gordon J.I. (2006). An obesity-associated gut microbiome with increased capacity for energy harvest. Nature.

[26] Million M., Angelakis E., Maraninchi M., Henry M., Giorgi R., Valero R., Vialettes B., Raoult D. (2013). Correlation between body mass index and gut concentrations of *Lactobacillus reuteri, Bifidobacterium animalis, Methanobrevibacter smithii and Escherichia*
*coli*. Int. J. Obes..

[27] Mack I., Cuntz U., Grmer C., Niedermaier S., Pohl C., Schwiertz A., Zimmermann K., Zipfel S., Enck P., Penders J. (2016). Weight gain in anorexia nervosa does not ameliorate the faecal microbiota, branched chain fatty acid profiles, and gastrointestinal complaints. Sci. Rep..

[28] Borgo F., Riva A., Benetti A., Casiraghi M.C., Bertelli S., Garbossa S., Anselmetti S., Scarone S., Pontiroli A.E., Morace G. (2017). Microbiota in anorexia nervosa: The triangle between bacterial species, metabolites and psychological tests. PLoS ONE.

[29] Armougom F., Henry M., Vialettes B., Raccah D., Raoult D. (2009). Monitoring bacterial community of human gut microbiota reveals an increase in *Lactobacillus* in obese patients and *Methanogens* in anorexic patients. PLoS ONE.

[30] Morita C., Tsuji H., Hata T., Gondo M., Takakura S., Kawai K., Yoshihara K., Ogata K., Nomoto K., Miyazaki K. (2015). Gut dysbiosis in patients with anorexia nervosa. PLoS ONE.

[31] Mörkl S., Lackner S., Müller W., Gorkiewicz G., Kashofer K., Oberascher A., Painold A., Holl A., Holzer P., Meinitzer A. (2017). Gut microbiota and body composition in anorexia nervosa inpatients in comparison to athletes, overweight, obese, and normal weight controls. Int. J. Eat. Disord..

[32] Mörkl S., Lackner S., Meinitzer A., Gorkiewicz G., Kashofer K., Painold A., Holl A., Holasek S. (2019). Pilot study: Gut microbiome and intestinal barrier in anorexia nervosa. Fortschr. Der Neurol. Psychiatr..

[33] Schulz N., Belheouane M., Dahmen B., Ruan V.A., Specht H.E., Dempfle A., Herpertz-Dahlmann B., Baines J.F., Seitz J. (2020). Gut microbiota alteration in adolescent anorexia nervosa does not normalize with short-term weight restoration. Int. J. Eat. Disord..

[34] Gutierrez E. (2013). A rat in the labyrinth of anorexia nervosa: Contributions of the activity-based anorexia rodent model to the understanding of anorexia nervosa. Int. J. Eat. Disord..

[35] Chowdhury T.G., Chen Y.W., Aoki C. (2015). Using the activity-based anorexia rodent model to study the neurobiological basis of anorexia nervosa. J. Vis. Exp..

[36] Casper R.C., Sullivan E.L., Tecott L. (2008). Relevance of animal models to human eating disorders and obesity. Psychopharmacology.

[37] Dixon D.P., Ackert A.M., Eckel L.A. (2003). Development of, and recovery from, activity-based anorexia in female rats. Physiol. Behav..

[38] Frintrop L., Liesbrock J., Paulukat L., Johann S., Kas M.J., Tolba R., Heussen N., Neulen J., Konrad K., Herpertz-Dahlmann B. (2018). Reduced astrocyte density underlying brain volume reduction inactivity-based anorexia rats. World J. Biol. Psychiatry.

[39] Epling W.F., Pierce W.D. (1988). Activity-based anorexia: A biobehavioral perspective. Int. J. Eat. Disord..

[40] Breton J., Legrand R., Achamrah N., Chan P., do Rego J.L., do Rego J.C., Coëffier M., Déchelotte P., Fetissov S.O. (2019). Proteome modifications of gut microbiota in mice with activity-based anorexia and starvation: Role in ATP production. Nutrition.

[41] Breton J., Tirelle P., Hasanat S., Pernot A., L’Huillier C., do Rego J.C., Déchelotte P., Coëffier M., Bindels L.B., Ribet D. (2021). Gut microbiota alteration in a mouse model of Anorexia Nervosa. Clin. Nutr..

[42] Queipo-Ortuño M.I., Seoane L.M., Murri M., Pardo M., Gomez-Zumaquero J.M., Cardona F., Casanueva F., Tinahones F.J. (2013). Gut Microbiota Composition in Male Rat Models under Different Nutritional Status and Physical Activity and Its Association with Serum Leptin and Ghrelin Levels. PLoS ONE.

[43] Trinh S., Kogel V., Voelz C., Schlösser A., Schwenzer C., Kabbert J., Heussen N., Clavel T., Herpertz-Dahlmann B., Beyer C. (2021). Gut microbiota ands brain alterations in a translational anorexia nervosa rat model. J. Psychiatr. Res..

[44] Chen J., Toyomasu Y., Hayashi Y., Linden D.R., Szurszewski J.H., Nelson H., Farrugia G., Kashyap P.C., Chia N., Ordog T. (2016). Altered gut microbiota in female mice with persistent low body weights following removal of post-weaning chronic dietary restriction. Genome Med..

[45] Dominique M., Legrand R., Galmiche M., Azhar S., Deroissart C., Guérin C., Do Rego J.L., Leon F., Nobis S., Lambert G. (2019). Changes in microbiota and bacterial protein caseinolytic peptidase b during food restriction in mice: Relevance for the onset and perpetuation of Anorexia Nervosa. Nutrients.

[46] Hata T., Miyata N., Takakura S., Yoshihara K., Asano Y., Kimura-Todani T., Yamashita M., Zhang X.T., Watanabe N., Mikami K. (2019). The Gut Microbiome Derived from Anorexia Nervosa Patients Impairs Weight Gain and Behavioral Performance in Female Mice. Endocrinology.

[47] Prochazkova P., Roubalova R., Dvorak J., Tlaskalova-Hogenova H., Cermakova M., Tomasova P., Sediva B., Kuzma M., Bulant J., Bilej M. (2019). Microbiota, microbial metabolites, and barrier function in a patient with anorexia nervosa after fecal microbiota transplantation. Microorganisms.

[48] De Clercq N.C., Frissen M.N., Davids M., Groen A.K., Nieuwdorp M. (2019). Weight Gain after Fecal Microbiota Transplantation in a Patient with Recurrent Underweight following Clinical Recovery from Anorexia Nervosa. Psychother. Psychosom..

[49] Jésus P., Ouelaa W., François M., Riachy L., Guérin C., Aziz M., Do Rego J.C., Déchelotte P., Fetissov S.O., Coëffier M. (2014). Alteration of intestinal barrier function during activity-based anorexia in mice. Clin. Nutr..

[50] Monteleone P., Carratù R., Cartenì M., Generoso M., Lamberti M., De Magistris L., Brambilla F., Colurcio B., Secondulfo M., Maj M. (2004). Intestinal permeability is decreased in anorexia nervosa. Mol. Psychiatry.

[51] Seitz J., Dahmen B., Keller L., Herpertz-Dahlmann B. (2020). Gut Feelings: How Microbiota Might Impact the Development and Course of Anorexia Nervosa. Nutrients.

[52] Gibson D., Mehler P.S. (2019). Anorexia Nervosa and the Immune System—A Narrative Review. J. Clin. Med..

[53] Bambury A., Sandhu K., Cryan J.F., Dinan T.G. (2018). Finding the needle in the haystack: Systematic identification of psychobiotics. Br. J. Pharmacol..

[54] Palmblad J., Fohlin L., Lundstrom M. (2009). Anorexia Nervosa and Polymorphonuclear (PMN) Granulocyte Reactions. Scand. J. Haematol..

[55] Gotch F.M., Spry C.J.F., Mowat A.G., Beeson P.B., Maclennan I.C. (1975). Reversible granulocyte killing defect in anorexia nervosa. Clin. Exp. Immunol..

[56] Dalton B., Bartholdy S., Robinson L., Solmi M., Ibrahim M.A.A., Breen G., Schmidt U., Himmerich H. (2018). A meta-analysis of cytokine concentrations in eating disorders. J. Psychiatr. Res..

[57] Solmi M., Veronese N., Favaro A., Santonastaso P., Manzato E., Sergi G., Correll C.U. (2015). Inflammatory cytokines and anorexia nervosa: A meta-analysis of cross-sectional and longitudinal studies. Psychoneuroendocrinology.

[58] Vaisman N., Hahn T. (1991). Tumor necrosis factor-α and anorexia-Cause or effect?. Metabolism.

[59] Allende L.M., Corell A., Manzanares J., Madruga D., Marcos A., Madroño A., López-Goyanes A., García-Pérez M.A., Moreno J.M., Rodrigo M. (1998). Immunodeficiency associated with anorexia nervosa is secondary and improves after refeeding. Immunology.

[60] Dalton B., Campbell I., Chung R., Breen G., Schmidt U., Himmerich H. (2018). Inflammatory Markers in Anorexia Nervosa: An Exploratory Study. Nutrients.

[61] Dalton B., Leppanen J., Campbell I.C., Chung R., Breen G., Schmidt U., Himmerich H. (2020). A longitudinal analysis of cytokines in anorexia nervosa. Brain. Behav. Immun..

[62] Patsalos O., Dalton B., Himmerich H. (2020). Effects of IL-6 signaling pathway inhibition on weight and BMI: A systematic review and meta-analysis. Int. J. Mol. Sci..

[63] Fink S., Eckert E., Mitchell J., Crosby R., Pomeroy C. (1996). T-lymphocyte subsets in patients with abnormal body weight: Longitudinal studies in anorexia nervosa and obesity. Int. J. Eat. Disord..

[64] Mustafa A., Ward A., Treasure J., Peakman M. (1997). T lymphocyte subpopulations in anorexia nervosa and refeeding. Clin. Immunol. Immunopathol..

[65] Elegido A., Graell M., Andrés P., Gheorghe A., Marcos A., Nova E. (2017). Increased naive CD4+ and B lymphocyte subsets are associated with body mass loss and drive relative lymphocytosis in anorexia nervosa patients. Nutr. Res..

[66] Nagata T., Kiriike N., Tobitani W., Kawarada Y., Matsunaga H., Yamagami S. (1999). Lymphocyte subset, lymphocyte proliferative response, and soluble interleukin-2 receptor in anorexic patients. Biol. Psychiatry.

[67] Wyatt R.J., Farrell M., Berry P.L., Forristal J., Maloney M.J., West C.D. (1982). Reduced alternative complement pathway control protein levels in anorexia nervosa: Response to parenteral alimentation. Am. J. Clin. Nutr..

[68] Brown R.F., Bartrop R., Beumont P., Birmingham C.L. (2005). Bacterial infections in anorexia nervosa: Delayed recognition increases complications. Int. J. Eat. Disord..

[69] Brown R.F., Bartrop R., Birmingham C.L. (2008). Immunological disturbance and infectious disease in anorexia nervosa: A review. Acta Neuropsychiatr..

[70] Belmonte L., Achamrah N., Nobis S., Guérin C., Riou G., Bôle-Feysot C., Boyer O., Richard V., Do Rego J.C., Déchelotte P. (2016). A role for intestinal TLR4-driven inflammatory response during activity-based anorexia. Sci. Rep..

[71] Miller A.H., Maletic V., Raison C.L. (2009). Inflammation and Its Discontents: The Role of Cytokines in the Pathophysiology of Major Depression. Biol. Psychiatry.

[72] Matcovitch-Natan O., Winter D.R., Giladi A., Aguilar S.V., Spinrad A., Sarrazin S., Ben-Yehuda H., David E., González F.Z., Perrin P. (2016). Microglia development follows a stepwise program to regulate brain homeostasis. Science.

[73] Reis W.L., Yi C.-X., Gao Y., Tschöp M.H., Stern J.E. (2015). Brain Innate Immunity Regulates Hypothalamic Arcuate Neuronal Activity and Feeding Behavior. Endocrinology.

[74] Collu R., Post J.M., Scherma M., Giunti E., Fratta W., Lutz B., Fadda P., Bindila L. (2020). Altered brain levels of arachidonic acid-derived inflammatory eicosanoids in a rodent model of anorexia nervosa. Biochim. Biophys. Acta Mol. Cell Biol. Lipids.

[75] Layé S. (2010). Polyunsaturated fatty acids, neuroinflammation and well being. Prostaglandins Leukot. Essent. Fat. Acids.

[76] Reyes-Ortega P., Ragu Varman D., Rodríguez V.M., Reyes-Haro D. (2020). Anorexia induces a microglial associated pro-inflammatory environment and correlates with neurodegeneration in the prefrontal cortex of young female rats. Behav. Brain Res..

[77] Seitz J., Konrad K., Herpertz-Dahlmann B. (2018). Extend, Pathomechanism and Clinical Consequences of Brain Volume Changes in Anorexia Nervosa. Curr. Neuropharmacol..

[78] Simon J.J., Stopyra M.A., Mönning E., Sailer S., Lavandier N., Kihm L.P., Bendszus M., Preissl H., Herzog W., Friederich H.C. (2020). Neuroimaging of hypothalamic mechanisms related to glucose metabolism in anorexia nervosa and obesity. J. Clin. Investig..

[79] Fonville L., Giampietro V., Williams S.C.R., Simmons A., Tchanturia K. (2014). Alterations in brain structure in adults with anorexia nervosa and the impact of illness duration. Psychol. Med..

[80] Frank G.K.W. (2014). Advances from neuroimaging studies in eating disorders. CNS Spectr..

[81] Florent V., Baroncini M., Jissendi-Tchofo P., Lopes R., Vanhoutte M., Rasika S., Pruvo J.-P., Vignau J., Verdun S., Johansen J.E. (2020). Hypothalamic Structural and Functional Imbalances in Anorexia Nervosa. Neuroendocrinology.

[82] Nilsson I.A.K. (2019). The ANX/ANX mouse—A valuable resource in anorexia nervosa research. Front. Neurosci..

[83] Nilsson I., Lindfors C., Fetissov S.O., Hökfelt T., Johansen J.E. (2008). Aberrant agouti-related protein system in the hypothalamus of theanx/anx mouse is associated with activation of microglia. J. Comp. Neurol..

[84] Nilsson I.A.K., Thams S., Lindfors C., Bergstrand A., Cullheim S., Hökfelt T., Johansen J.E. (2011). Evidence of hypothalamic degeneration in the anorectic anx/anx mouse. Glia.

[85] Frank G.K.W., Deguzman M.C., Shott M.E., Laudenslager M.L., Rossi B., Pryor T. (2018). Association of Brain Reward Learning Response with Harm Avoidance, Weight Gain, and Hypothalamic Effective Connectivity in Adolescent Anorexia Nervosa. JAMA Psychiatry.

[86] Jais A., Brüning J.C. (2017). Hypothalamic inflammation in obesity and metabolic disease. J. Clin. Investig..

[87] Stefaniak J., O’brien J. (2016). Imaging of neuroinflammation in dementia: A review. J. Neurol. Neurosurg. Psychiatry.

[88] Guertin T.L. (1999). Eating behavior of bulimics, self-identified binge eaters, and non-eating-disordered individuals: What differentiates these populations?. Clin. Psychol. Rev..

[89] Buchanan J.B., Johnson R.W. (2007). Regulation of food intake by inflammatory cytokines in the brain. Neuroendocrinology.

[90] Nakai Y., Hamagaki S., Takagi R., Taniguchi A., Kurimoto F. (2000). Plasma concentrations of tumor necrosis factor-α (TNF-α) and soluble TNF receptors in patients with bulimia nervosa. Clin. Endocrinol..

[91] MacDowell K.S., Díaz-Marsá M., Güemes I., Rodríguez A., Leza J.C., Carrasco J.L. (2013). Inflammatory activation and cholinergic anti-inflammatory system in eating disorders. Brain Behav. Immun..

[92] Tabasi M., Anbara T., Siadat S.D., Khezerloo J.K., Elyasinia F., Bayanolhagh S., Safavi S.A.S., Yazdannasab M.R., Soroush A., Bouzari S. (2020). Socio-demographic Characteristics, Biochemical and Cytokine Levels in Bulimia Nervosa Candidates for Sleeve Gastrectomy. Arch. Iran. Med..

[93] Raymond N.C., Dysken M., Bettin K., Eckert E.D., Crow S.J., Markus K., Pomeroy C. (2000). Cytokine Production in Patients with Anorexia Nervosa, Bulimia Nervosa, and Obesity. Int. J. Eat. Disord..

[94] Opal A.S.M., Depalo V.A. (2000). Anti-Inflammatory Cytokines (*). Chest.

[95] Caroleo M., Carbone E.A., Greco M., Corigliano D.M., Arcidiacono B., Fazia G., Rania M., Aloi M., Gallelli L., Segura-Garcia C. (2019). Brain-behavior-immune interaction: Serum cytokines and growth factors in patients with eating disorders at extremes of the body mass index (bmi) spectrum. Nutrients.

[96] Brennan F.M., Green P., Amjadi P., Robertshaw H.J., Alvarez-Iglesias M., Takata M. (2008). Interleukin-10 regulates TNF-α-converting enzyme (TACE/ADAM-17) involving a TIMP-3 dependent and independent mechanism. Eur. J. Immunol..

[97] Pestka S., Krause C.D., Sarkar D., Walter M.R., Shi Y., Fisher P.B. (2004). Interleukin-10 and related cytokines and receptors. Annu. Rev. Immunol..

[98] Shank L.M., Tanofsky-Kraff M., Kelly N.R., Schvey N.A., Marwitz S.E., Mehari R.D., Brady S.M., Demidowich A.P., Broadney M.M., Galescu O.A. (2017). Pediatric Loss of Control Eating and High-Sensitivity C-Reactive Protein Concentrations. Child. Obes..

[99] Succurro E., Segura-Garcia C., Ruffo M., Caroleo M., Rania M., Aloi M., De Fazio P., Sesti G., Arturi F. (2015). Obese patients with a binge eating disorder have an unfavorable metabolic and inflammatory profile. Medicine.

[100] Brandão P.P., Garcia-Souza E.P., Neves F.A., Jose M., Sichieri R., De Moura E.G., Cristina P., Moura A.S. (2010). Leptin/adiponectin ration in obese women with and without binge eating disorder. Neuro Endocrinol. Lett..

[101] Blomquist K.K., Milsom V.A., Barnes R.D., Boeka A.G., White M.A., Masheb R.M., Grilo C.M. (2012). Metabolic syndrome in obese men and women with binge eating disorder: Developmental trajectories of eating and weight-related behaviors. Compr. Psychiatry.

[102] Taylor A.E., Hubbard J., Anderson E.J. (1999). Impact of binge eating on metabolic and leptin dynamics in normal young women. J. Clin. Endocrinol. Metab..

[103] Kral J.G., Buckley M.C., Kissileff H.R., Schaffner F. (2001). Metabolic correlates of eating behavior in severe obesity. Int. J. Obes..

[104] Marcos A., Varela P., Santacruz A., Munoz-Velez A., Morande G. (1993). Nutritional status and immunocompetence in eating disorders. A comparative study. Eur. J. Clin. Nutr..

[105] Marcos A., Varela P., Toro O., Nova E., Lopez-Vidriero I., Morande G. (1997). Evaluation of nutritional status by immunologic assessment in bulimia nervosa: Influence of body mass index and vomiting episodes. Am. J. Clin. Nutr..

[106] Zerwas S., Larsen J.T., Petersen L., Thornton L.M., Quaranta M., Koch S.V., Pisetsky D., Mortensen P.B., Bulik C.M. (2017). Eating disorders, autoimmune, and autoinflammatory disease. Pediatrics.

[107] Raevuori A., Haukka J., Vaarala O., Suvisaari J.M., Gissler M., Grainger M., Linna M.S., Suokas J.T. (2014). The increased risk for autoimmune diseases in patients with eating disorders. PLoS ONE.

[108] Yilmaz Z., Gottfredson N.C., Zerwas S.C., Bulik C.M., Micali N. (2019). Developmental Premorbid Body Mass Index Trajectories of Adolescents With Eating Disorders in a Longitudinal Population Cohort. J. Am. Acad. Child Adolesc. Psychiatry.

[109] Corcos M., Guilbaud O., Paterniti S., Moussa M., Chambry J., Chaouat G., Consoli S.M., Jeammet P. (2003). Involvement of cytokines in eating disorders: A critical review of the human literature. Psychoneuroendocrinology.

[110] Harrison N.A., Voon V., Cercignani M., Cooper E.A., Pessiglione M., Critchley H.D. (2016). A neurocomputational account of how inflammation enhances sensitivity to punishments versus rewards. Biol. Psychiatry.

[111] Cifani C., Polidori C., Melotto S., Ciccocioppo R., Massi M. (2009). A preclinical model of binge eating elicited by yo-yo dieting and stressful exposure to food: Effect of sibutramine, fluoxetine, topiramate, and midazolam. Psychopharmacology.

[112] Cifani C., Di Bonaventura M.V.M., Ciccocioppo R., Massi M., Avena N.M. (2013). Binge Eating in Female Rats Induced by Yo-Yo Dieting and Stress. Animal Models of Eating Disorders. Neuromethods.

[113] Alboni S., Micioni Di Bonaventura M.V., Benatti C., Giusepponi M.E., Brunello N., Cifani C. (2017). Hypothalamic expression of inflammatory mediators in an animal model of binge eating. Behav. Brain Res..

[114] Zorrilla E.P., Conti B. (2014). Interleukin-18 null mutation increases weight and food intake and reduces energy expenditure and lipid substrate utilization in high-fat diet fed mice. Brain. Behav. Immun..

[115] Netea M.G., Joosten L.A.B., Lewis E., Jensen D.R., Voshol P.J., Kullberg B.J., Tack C.J., Van Krieken H., Kim S.H., Stalenhoef A.F. (2006). Deficiency of interleukin-18 in mice leads to hyperphagia, obesity and insulin resistance. Nat. Med..

[116] Green S.J., Scheller L.F., Marletta M.A., Seguin M.C., Klotz F.W., Slayter M., Nelson B.J., Nacy C.A. (1994). Nitric oxide: Cytokine-regulation of nitric oxide in host resistance to intracellular pathogens. Immunol. Lett..

[117] Morley J.E., Farr S.A., Sell R.L., Hileman S.M., Banks W.A. (2011). Nitric oxide is a central component in neuropeptide regulation of appetite. Peptides.

[118] Vannacci A., Ravaldi C., Giannini L., Rotella C.M., Masini E., Faravelli C., Ricca V. (2006). Increased nitric oxide production in eating disorders. Neurosci. Lett..

